# The Dynamics of Attention during Free Looking

**DOI:** 10.1371/journal.pone.0056428

**Published:** 2013-02-14

**Authors:** Sarah Enos Watamura, Katie A. Devine, Steven S. Robertson

**Affiliations:** 1 Department of Psychology, University of Denver, Denver, Colorado, United States of America; 2 Department of Radiation Oncology, University of Rochester Medical Center, Rochester, New York, United States of America; 3 Department of Human Development, Cornell University, Ithaca, New York, United States of America; Queensland Brain Institute, Australia

## Abstract

Simple methods to study attention dynamics in challenging research and practical applications are limited. We explored the utility of examining attention dynamics during free looking with steady-state visual evoked potentials (SSVEPs), which reflect the effects of attention on early sensory processing. This method can be used with participants who cannot follow verbal instructions and patients without voluntary motor control. In our healthy participants, there were robust fluctuations in the strength of SSVEPs driven by the fixated and non-fixated stimuli (rapidly changing pictures of faces) in the seconds leading up to the moment they chose to shift their gaze to the next stimulus sequence. Furthermore, the amplitude of SSVEPs driven by the fixated stimuli predicted subsequent recognition of individual stimuli. The results illustrate how information about the temporal course of attention during free looking can be obtained with simple methods based on the attentional modulation of SSVEPs.

## Introduction

Tracking changes in attention independent of eye or body movements has high potential utility for a number of challenging practical and research applications. For example, brain-computer interfaces for use with patients who are unable to voluntarily control their movements allow a computer to receive instructions directly from measured brain activity [1]. Similarly, in research applications utilizing participants who cannot reliably follow instructions [2], or in situations where providing instructions would compromise the study aims [3], simple methods that permit the dynamics of attention during free looking to be monitored independently of eye gaze allow previously unexplored questions to be addressed. Key requirements for these applications include the need for a strong and reliable brain signal that reflects rapid changes in attention, relative ease of recording without cumbersome equipment, and simple display properties. Steady-state visual evoked potentials (SSVEPs) appear to meet these requirements.

The steady-state visual evoked potential is a continuous oscillation in brain electrical potential driven by a periodic (flickering) visual stimulus [4], [5]. Oscillations at the fundamental and harmonic frequencies of the stimulus can be detected across wide areas of the scalp [6], [7], but are most prominent in posterior regions over visual cortex where they are generated [8]–[10]. Importantly, spatial attention modulates the amplitude and sometimes phase of SSVEPs recorded over visual cortex [11]–[18], making them particularly useful indicators of attention dynamics [18]–[20]. For example, the amplitude of the SSVEP is greater when a flickering stimulus is attended (with no change in gaze) compared to when it is not attended. The neural mechanisms underlying the attentional modulation of SSVEPs are not fully understood, but likely include changes in contrast gain and other top-down effects of attention in early visual areas [13], [14], [17], [18], [21].

The ability to track the dynamics of attention by examining the temporal course of changes in SSVEP amplitude and phase in relatively unconstrained conditions has clear benefits. For example, the attentional modulation of SSVEPs has recently been exploited in brain-computer interfaces, where the relative amplitude of SSVEPs driven by targets flickering at different frequencies has been used to implement virtual pointing operations [1], [22]. One goal of work in this area is to develop brain-computer interfaces that support communication and action by individuals with motor impairments, including lack of oculomotor control [23]–[24].

In the present study, our specific aim was to demonstrate the utility of SSVEPs for studying attention dynamics when participants shift their gaze at moments of their own choosing, using very simple presentation and recording techniques. Gaze shifts are critical points in attention dynamics, when one source of visual information may be abandoned and another more fully engaged [25]–[32]. We used SSVEPs to examine changes in spatial attention during the final seconds of prolonged inspections of a target before a *spontaneous* (un-cued) gaze shift to another target. Participants were presented simultaneously with sequences of rapidly changing stimuli at two locations while we recorded brain activity over extrastriate cortex. After careful inspection of the stimulus sequence at one location, participants shifted their gaze *at moments of their own choosing* to the stimulus sequence at the other location. Our objective was to document the temporal course of changes in spatial attention to both the fixated and non-fixated stimuli, as reflected in SSVEP amplitude and phase, during the seconds preceding spontaneous (un-cued) gaze shifts.

## Methods

### Ethics Statement

This study was approved by the Cornell University Institutional Review Board (protocol # 9201002). Written, informed consent was obtained from all participants.

### Participants

Twenty young adults (19–21 years, 16 female) with normal or corrected-to-normal vision, recruited from undergraduate classes, served as volunteer participants. The data from an additional six participants could not be used because of excessive body movement (repeated fidgeting or postural adjustments that interfered with the experimenter's ability to monitor gaze, n = 3), sleepiness (repeated eye closures other than blinks, (n = 2), or experimenter error (n = 1).

### Stimuli

**Figure 1:**
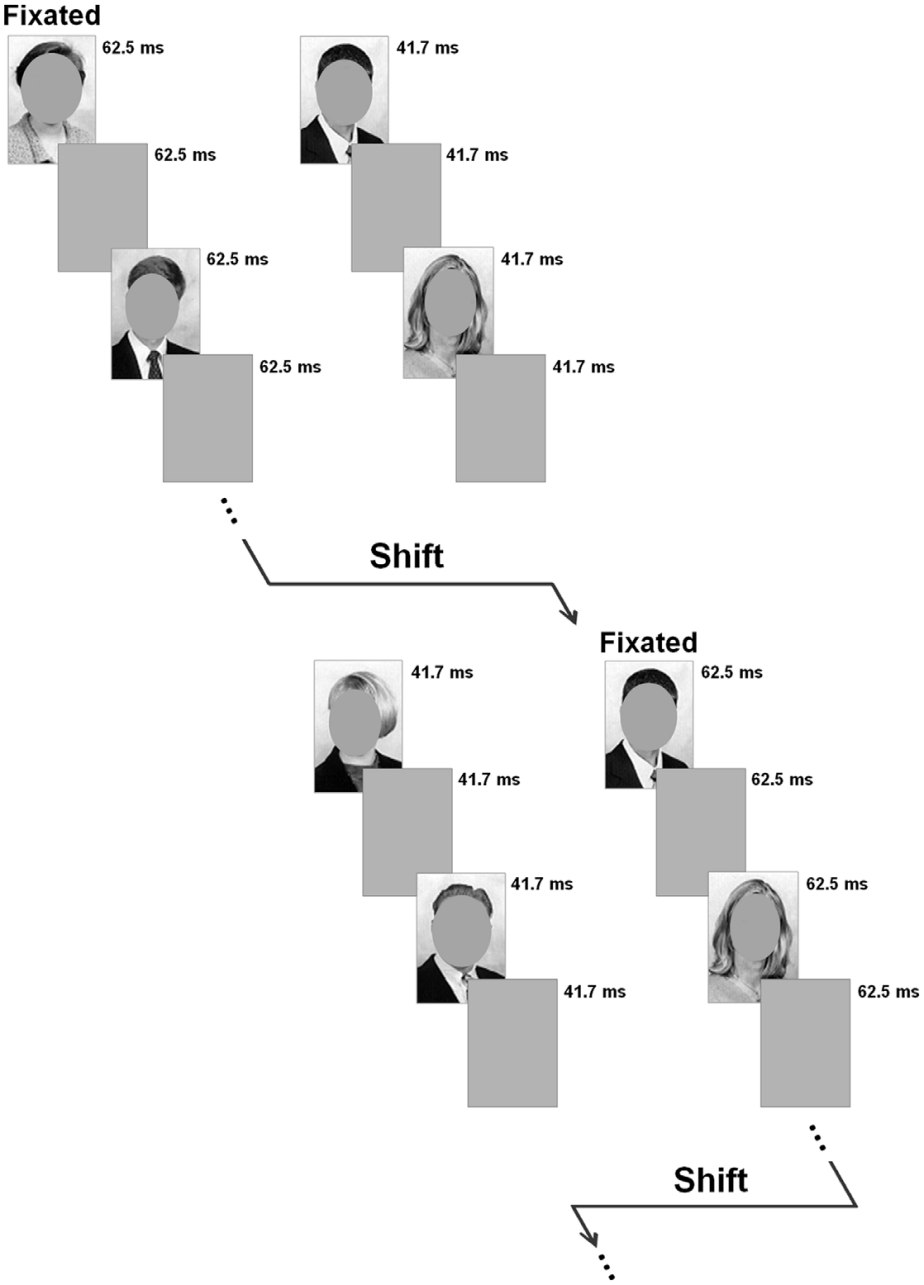
Stimulus sequences preceding un-cued gaze shifts (one shift set). Stimuli were 1.8×2.8 cm (3.4×5.3 deg visual angle). Faces were not covered in the experiment. Two stimulus sequences were displayed simultaneously on a black background, separated horizontally by 4.2 cm (8.0 deg visual angle). Each sequence consisted of repeated presentations of five different stimuli in a random order. A homogeneous gray rectangle was displayed between successive stimuli for the same duration as the stimuli. The stimuli in the fixated sequence changed at 8 Hz, and the stimuli in the non-fixated sequence changed at 12 Hz (the rates for the left and right stimulus sequences switched when gaze shifted so the fixated sequence always changed at 8 Hz). Participants were instructed to look at the stimulus sequence on the starting side (left or right, counterbalanced across participants). At a time of their own choosing, they were to shift their gaze to look at the other, previously non-fixated, stimulus sequence. At each shift, the previously fixated stimulus sequence was replaced. They continued in this manner, alternating sides, for three sets of two shifts (one shift set is shown), after which there was a brief rest period during which recognition of the stimuli was tested. Each participant completed five blocks of three shift sets. Details for the stimuli and procedure are in the text.

The stimuli were 300 gray scale portrait photographs (162 female) scanned from college yearbooks from the early 1990's. See Figure 1. Portrait photographs were used to enhance participants' interest in the task. Yearbook photographs were used so that pose, lighting, and other visual characteristics of the photographs would be relatively similar; they all showed the person's head and shoulders on a light neutral background. When cropped and displayed on a monitor at 32 pixels/cm they were 1.8 cm wide×2.8 cm high (3.4×5.3 deg visual angle). The display background was black, and the front of the monitor was covered with black cloth that had a rectangular window (11.0 cm wide×5.5 cm high) through which the stimuli were visible.

Two stimulus sequences were displayed simultaneously, separated horizontally by 4.2 cm (8.0 deg visual angle). Each sequence consisted of repeated presentations of five different stimuli in a random order (with the constraint that a stimulus could not follow itself) so there would be no correlation between time and the presentation of specific stimuli. A homogeneous gray rectangle the same size as the stimuli was displayed between successive stimuli for the same duration as the stimuli. The luminance of the gray rectangle (33 cd/m^2^) was chosen to match the mean space-average luminance of the stimuli to minimize distraction and fatigue in the participants.

The stimuli in the fixated sequence changed at 8 Hz, and the stimuli in the non-fixated sequence changed at 12 Hz (i.e., the rates for the left and right stimulus sequences switched when gaze shifted so the fixated sequence always changed at 8 Hz; see Procedure, below). Stimulus flicker frequencies between 8 and 12 Hz have previously been shown to elicit attention-modulated SSVEPs that are detectable over relatively broad regions of occipital-temporal cortex [14], [15], [19]. The pulse trains controlling the stimulus sequences were generated by hardware counters and acquired at 256 Hz along with the EEG. The 60 Hz refresh rate of the display therefore introduced ± 8.3 ms jitter to alternate picture onset times in the 8 Hz stimulus sequence. Although the sequence completed exactly two cycles in each 250 ms analysis window, the jitter likely made the SSVEP less robust. The 12 Hz sequence was not affected by the display refresh rate.

### Procedure

Participants sat in a chair with their chin resting lightly on a padded surface that was adjusted to a comfortable height. Their eyes were approximately 30 cm from the monitor that displayed the stimuli. They were instructed to look at the stimulus sequence on the starting side (left or right) to familiarize themselves with the five individual stimuli. See Figure 1. At a time of their own choosing, they were to shift their gaze to look at the other, previously non-fixated, stimulus sequence. The previously fixated stimulus sequence was replaced immediately after the participant looked away from it by the experimenter watching the video camera that was recording gaze for later off-line analysis (see Gaze Shift Timing, below). They were to continue in this manner, alternating sides, for three sets of two shifts (each shift set included one shift from each side), after which there was a brief rest period. Each participant completed five blocks of three shift sets, which allowed participants to remain actively engaged in the task without excessive fatigue. Starting side was counterbalanced across participants. After the task was explained, participants completed one or two practice blocks before beginning the experiment.

To increase participants' motivation to attend to the individual stimuli and to obtain inspections that were long enough to permit the planned SSVEP analyses (see below), participants were told that there would be a recognition test at the end of each block. For the test, they were shown a card with three stimuli and asked, “Which of these pictures do you recognize?” Given how quickly the pictures in a sequence changed and the subjective experience that they were flashing too quickly to encode, two of the three stimuli presented in the recognition test had been among the 30 stimuli displayed during the block in order to increase the likelihood that participants would recognize at least one. Performance on the recognition tests was measured by the signal detection sensitivity index, *d′* [33].

A total of 12 to 30 (median = 18) shifts per participant were analyzed. Blocks of shifts were excluded if there was excessive body movement (fidgeting or postural adjustments that interfered with the experimenter's ability to monitor gaze, n = 4), sleepiness (eye closure other than blinks, n = 1), distraction (self-report, n = 4; audible telephone ring, n = 1), looking away from stimulus sequences (n = 15), or experimenter error/equipment malfunction (n = 6). Blocks containing a look duration less than 5 s (n = 5) were also excluded.

### EEG

Brain electrical potentials were recorded with pre-gelled, disposable Ag-AgCl electrodes (Conmed 1620) on the occipital-temporal scalp. To minimize preparation time and distraction for the participants, only two signal electrodes were used. The signal electrodes were placed at TO1 and TO2, based on a modified 10–20 system [34]. TO1 and TO2 are sites at which strong attentional modulation of SSVEPs has been documented [14], [18]. The reference electrode was placed 3 cm directly above the inion. The ground electrode was placed 3 cm to the right of the reference electrode. This placement [35] allowed all of the electrodes to be mounted in a comfortable elasticized cloth headband that held the electrodes firmly against the scalp. Hair was moved away from the electrode sites, but the scalp was not cleaned or abraded. Electrode impedances were less than 10 kΩ.

EEG signals were amplified with a gain of 100,000 and band-pass filtered with cut-off (−6 dB) frequencies of 1 and 100 Hz; a notch filter was used to reduce residual 60 Hz noise (Grass Model 15 Neurodata Amplifier System, input impedance 20 MΩ). The amplified and filtered signals were digitized on-line at 256 samples/s with 12 bit resolution between ±10 V (National Instruments PCI-6023E), corresponding to a resolution of 0.05 µV for the EEG signals.

### Gaze Shift Timing

Participants' gaze was recorded (Panasonic AG-7350) for later off-line analysis with a video camera (Cohu 4910) centered above the stimulus monitor. The off-line video analysis was done by two independent coders who determined the timing of each gaze shift to the nearest video field (17 ms) by examining the corneal reflections of the stimuli in each field of the video record. Based on 180 shifts (9 consecutive shifts from each participant), the two coders' determinations differed by no more than one video field for 98 percent of the shifts, and never differed by more than 3 video fields (50 ms). When the independent determinations differed, the two coders jointly reexamined the video record to obtain a consensus value for the timing of the gaze shift.

The off-line analysis of the video record was used to define the 4 s SSVEP analysis period preceding each shift (see SSVEP Analysis, below). The off-line video analysis also confirmed that the replacement of the previously fixated stimulus sequence following each gaze shift during the experiment (see Procedure, above) occurred before the beginning of the 4 s SSVEP analysis period for all shifts and participants in the final sample.

### SSVEP Analysis

**Figure 2:**
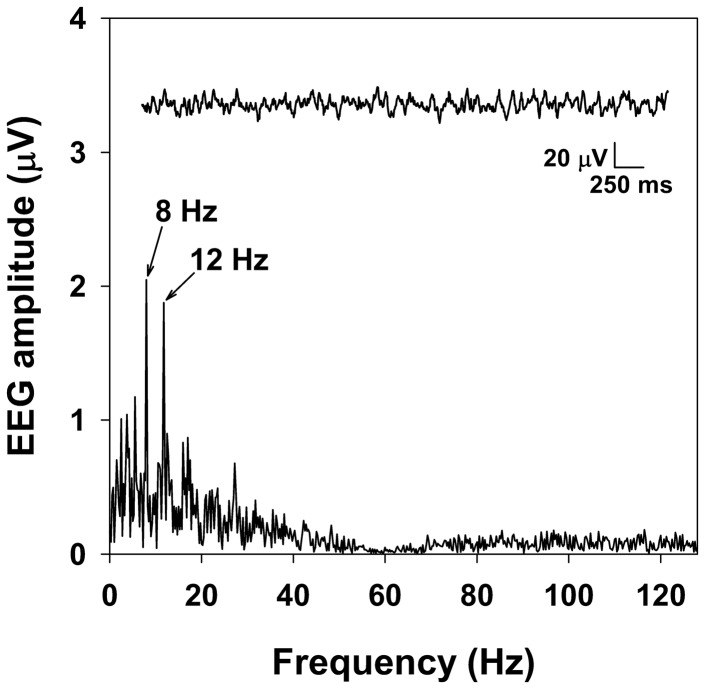
Amplitude spectrum of EEG. Example of EEG (inset) recorded at TO1 from one participant during the 4 s before an un-cued gaze shift from the stimulus sequence on the left to the stimulus sequence on the right (see Figure 1). Peaks in the amplitude spectrum are evident at the flicker frequencies of the fixated (8 Hz) and non-fixated (12 Hz) stimulus sequences.

EEG was analyzed off-line using the Fast Fourier Transform implemented in LabVIEW (ver. 5.1, National Instruments), time-locked to gaze shifts as determined by the off-line, field-by-field analysis of the video record (see Gaze Shift Timing, above). See Figure 2. SSVEP amplitude (µV) at 8 and 12 Hz, phase delay (timing of peak SSVEP amplitude relative to stimulus onset, expressed as a fraction of the stimulus cycle), and phase locking independent of amplitude [36] calculated across blocks, were determined in a 250 ms moving window every 31.25 ms during the 4 s preceding each gaze shift in usable blocks. SSVEP amplitude at higher harmonics of the stimulus frequencies was negligible. Because of the contralateral representation of the left and right visual hemifields in visual cortex, EEG recorded at TO1 was used to measure SSVEPs elicited by both stimulus sequences when the participant fixated the left sequence, and TO2 was used when the participant fixated the right sequence.

SSVEP amplitude, phase delay, and phase locking during the 4 s before gaze shifted were submitted to Stimulus (fixated, non-fixated)×Lead (TO1, TO2)×Shift Set (1–3)×Time (121 windows) analyses of variance, with repeated measures on all factors. Due to the small number of males in the final sample, gender was not analyzed. The two shifts within each shift set from usable blocks were averaged before the analyses of variance were conducted.

## Results

**Figure 3:**
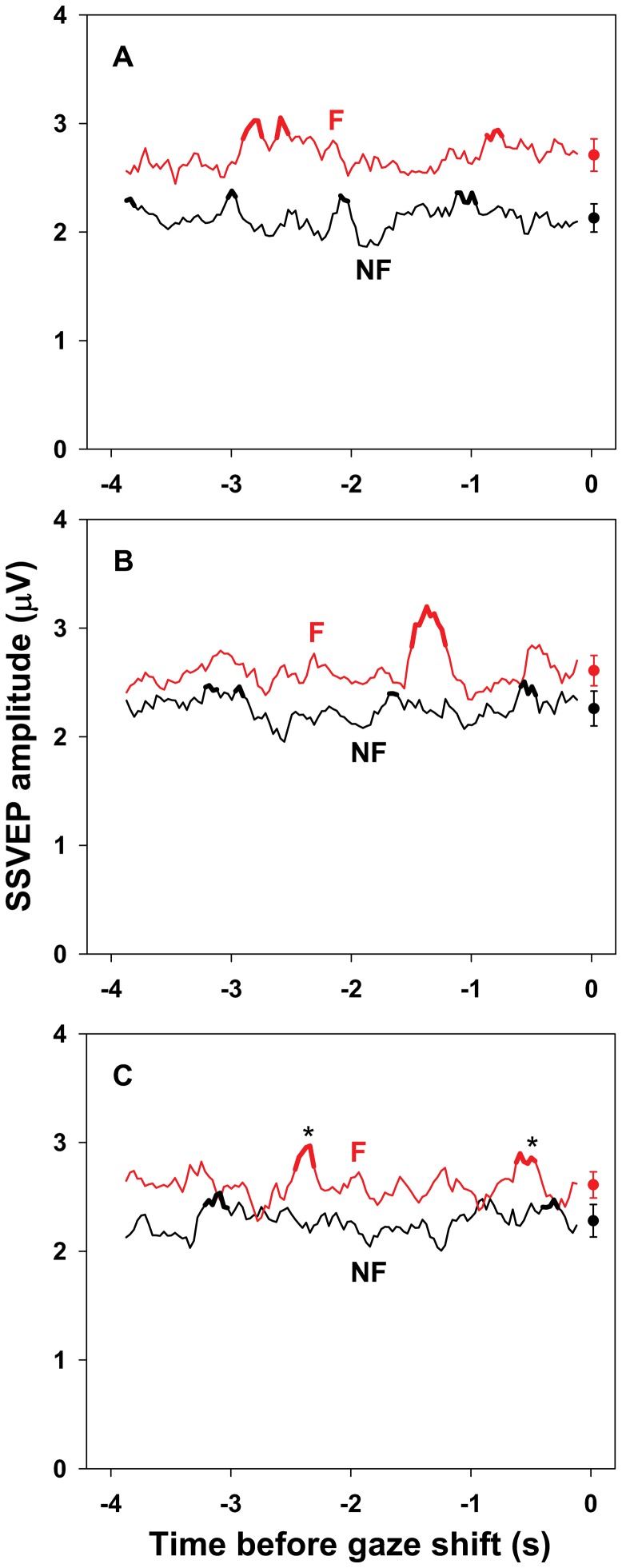
SSVEP amplitude preceding un-cued gaze shifts. Mean amplitude (µV) of SSVEPs elicited by the fixated (F, red curve) and non-fixated (NF, black curve) stimulus sequences in a 250 ms moving window during the 4 s preceding un-cued gaze shifts in the first (A), second (B), and third (C) shift sets. Standard errors for each moving window have been omitted to reduce visual clutter. The mean (± SEM) SSVEP amplitude for each shift set is plotted to the right of the corresponding time series. The decreased difference between SSVEP amplitude driven by the fixated and non-fixated stimulus sequences after the first shift set (fixated 2.71±.15 vs. non-fixated 2.13±0.13 for shift set 1, 2.61±.14 vs. 2.26±0.16 for shift set 2, 2.61±.12 vs. 2.28±0.15 for shift set 3; mean ± SEM µV), indicated by a Stimulus×Shift Set interaction (*p* = .003) in the analysis of variance, is evident. The dependence of this trend on the time before the next shift, indicated by a Stimulus×Shift Set×Time interaction (*p* = .008), is also evident. The thick portions of the curves indicate times during which SSVEP amplitude exceeded 1 SD above the mean for more than 2 consecutive windows. Asterisks (*) mark the local maxima within those intervals for which SSVEP amplitude predicted subsequent recognition of fixated stimuli as measured by *d′* (Pearson correlation, *p*<.0005). Details of the analyses are in the text.

**Figure 4:**
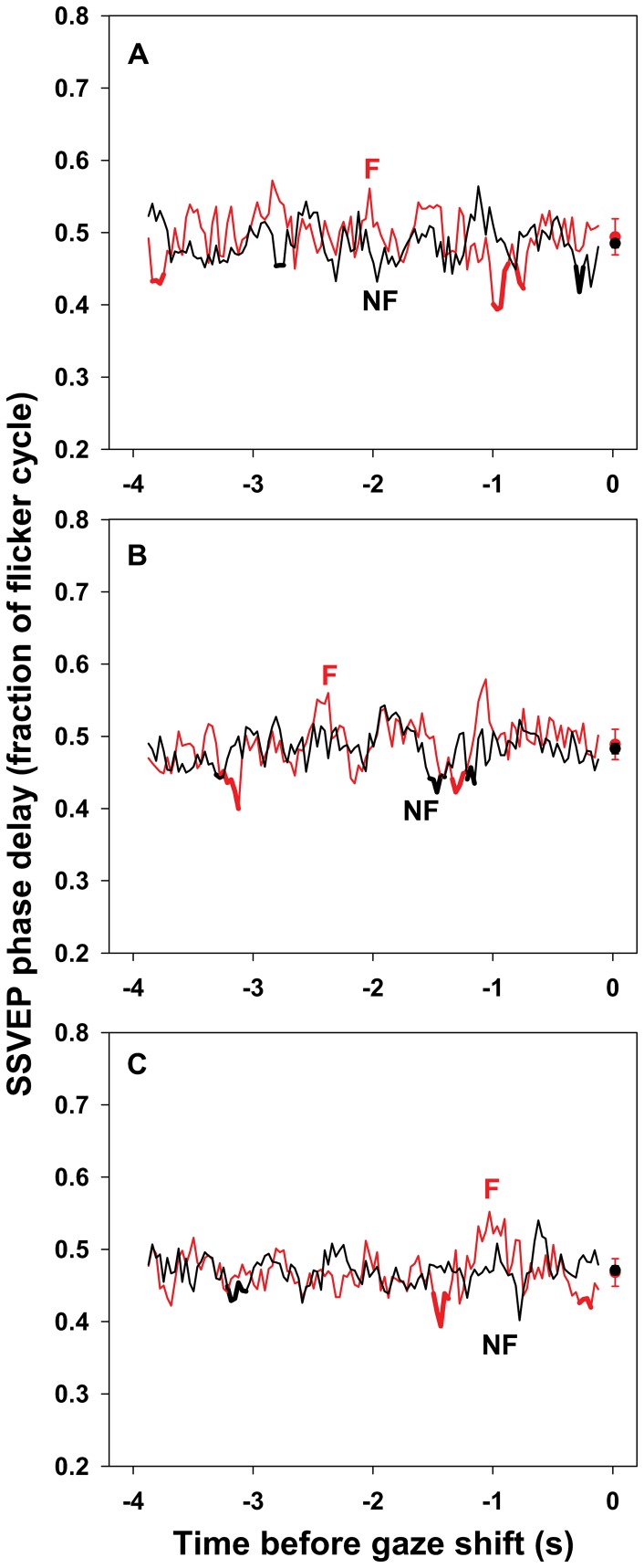
SSVEP phase delay preceding un-cued gaze shifts. Mean phase delay of SSVEPs elicited by the fixated (F, red curve) and non-fixated (NF, black curve) stimulus sequences in a 250 ms moving window during the 4 s preceding un-cued gaze shifts in the first (A), second (B), and third (C) shift sets. SSVEP phase delay is the timing of peak SSVEP amplitude relative to stimulus onset, expressed as a fraction of the stimulus cycle. Standard errors for each moving window have been omitted to reduce visual clutter. The mean (± SEM) SSVEP phase delay for each shift set is plotted to the right of the corresponding time series. The slight decrease in the average phase delay in the third shift set (0.490±0.013, 0.486±0.012, and 0.470±0.010 cycle in shift sets 1, 2, and 3, respectively; mean ± SEM), indicated by a main effect of Shift Set (*p* = .037), is evident. The thick portions of the curves indicate times during which phase delay was more than 1 SD below the mean for more than 2 consecutive windows. Local minima within those intervals did not predict subsequent recognition of fixated stimuli as measured by *d′*. Details of the analyses are in the text.

**Figure 5:**
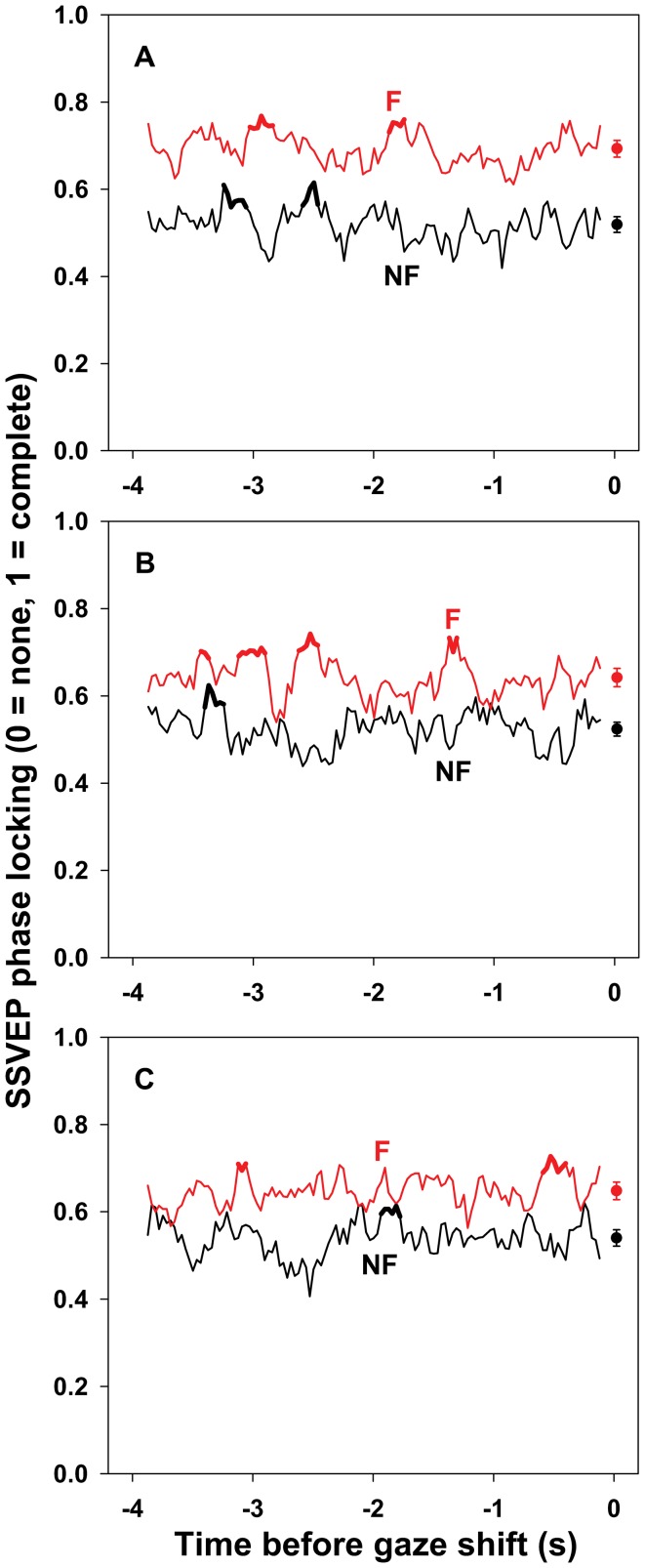
SSVEP phase locking preceding un-cued gaze shifts. Mean phase locking (0, no phase locking; 1, complete phase locking) of SSVEPs to the fixated (F, red curve) and non-fixated (NF, black curve) stimulus sequences in a 250 ms moving window during the 4 s preceding un-cued gaze shifts in the first (A), second (B), and third (C) shift sets. Standard errors for each moving window have been omitted to reduce visual clutter. The mean (± SEM) phase locking to each stimulus sequence for each shift set is plotted to the right of the corresponding time series. The decreased difference between phase locking to the fixated and non-fixated stimulus sequences after the first shift set (fixated 0.693±0.019 vs. non-fixated 0.519±0.018 for shift set 1, 0.642±0.021 vs. 0.524±0.016 for shift set 2, 0.648±0.020 vs. 0.540±0.019 for shift set 3; mean ± SEM), indicated by a Stimulus×Shift Set interaction (*p* = .004) in the analysis of variance, is evident. The dependence of the difference between phase locking to the fixated and non-fixated stimuli on the time until the next shift, as indicated by a Stimulus×Time interaction (*p* = .036), is also evident. The thick portions of the curves indicate times during which phase locking exceeded 1 SD above the mean for more than 2 consecutive windows. Local maxima within those intervals did not predict subsequent recognition of fixated stimuli as measured by *d′*. Details of the analyses are in the text.

Because visual sensitivity varies with retinal eccentricity [37] and SSVEP amplitude depends on flicker frequency [15], simple differences between SSVEPs elicited by fixated and non-fixated stimuli are not interpretable. Therefore, although main effects of Stimulus are reported, only interactions of Stimulus with other factors are discussed. Where relevant, the reported *p*-values reflect the Huynh-Feldt correction for non-sphericity. Table 1 contains the mean (and SEM) SSVEP amplitude, phase delay, and phase locking for each combination of Stimulus, Lead, and Shift Set. Figures 3, 4, and 5 show the data relevant to interactions of these factors with Time.

**Table 1:** Mean (SEM) SSVEP amplitude, phase delay, and phase locking.

		SSVEP Amplitude		SSVEP Phase Delay		SSVEP Phase Locking	
Shift Set	Stimulus	TO1	TO2	TO1	TO2	TO1	TO2
1	Fixated	2.77 (0.19)	2.75 (0.16)	.472 (.036)	.516 (.028)	.697 (.030)	.689 (.024)
	Non-fixated	2.07 (0.13)	2.19 (0.15)	.486 (.008)	.485 (.010)	.524 (.020)	.514 (.021)
2	Fixated	2.58 (0.18)	2.64 (0.14)	.471 (.031)	.507 (.022)	.639 (.028)	.645 (.024)
	Non-fixated	2.22 (0.17)	2.31 (0.17)	.485 (.011)	.482 (.006)	.526 (.018)	.523 (.017)
3	Fixated	2.60 (0.17)	2.62 (0.10)	.441 (.028)	.496 (.023)	.660 (.025)	.636 (.027)
	Non-fixated	2.23 (0.16)	2.33 (0.16)	.466 (.011)	.475 (.005)	.548 (.204)	.532 (.016)

SSVEP amplitude (µV) and phase delay with respect to stimulus onset (fraction of stimulus interval) were averaged across blocks of shift sets, time intervals within shift sets, and participants. Phase locking to stimulus flicker (0, no phase locking; 1, complete phase locking) was calculated across blocks of shift sets and averaged across time intervals within shift sets and participants. Details of analyses are in the text. Relevant time series are shown in Figures 3, 4, and 5.

### SSVEP Amplitude

As expected, there was a main effect of Stimulus on SSVEP amplitude, *F*(1,19) = 11.52, *p* = .003, reflecting the larger amplitude elicited by the fixated compared to the non-fixated stimuli (2.64±0.13 vs. 2.22±0.14 µV, respectively).

However, the average difference between the amplitudes of the SSVEPs elicited by the fixated and non-fixated stimuli differed across shift sets, as indicated by a Stimulus×Shift Set interaction, *F*(2,38) = 7.77, *p* = .003. The average amplitude of the SSVEP elicited by the fixated stimuli decreased slightly after the first shift set (2.71±.15, 2.61±.14, and 2.61±.12 µV in shift sets 1, 2, and 3, respectively; mean ± SEM) and the average amplitude of the SSVEP elicited by the non-fixated stimuli increased slightly, also after the first shift set (2.13±0.13, 2.26±0.16, and 2.28±0.15 µV in shift sets 1, 2, and 3, respectively). See Figure 3.

Furthermore, the differential changes across shift sets in SSVEP amplitude elicited by the fixated and non-fixated stimuli depended strongly on the time until the next shift, as indicated by a Stimulus×Shift Set×Time interaction, *F*(240,4560) = 1.52, *p* = .008. That is, the temporal locations of large and small differences between the amplitude of the SSVEPs driven by the fixated and non-fixated stimuli varied across shift sets. See Figure 3. There were no other main or interaction effects for SSVEP amplitude, all *p*>.05.

### SSVEP Phase Delay

There were no main or interaction effects involving Stimulus on SSVEP phase delay, all *p*>.05. However, there was a main effect of Shift Set, *F*(2,38) = 4.10, *p* = .037, reflecting a slight decrease in phase delay in the third shift set (0.490±0.013, 0.486±0.012, and 0.470±0.010 cycle in shift sets 1, 2, and 3, respectively). See Figure 4. There were no other main or interaction effects for SSVEP phase delay, all *p*>.05.

### SSVEP Phase Locking

There was a main effect of Stimulus on SSVEP phase locking, *F*(1,19) = 58.07, *p*<.001, reflecting greater phase locking to the fixated compared to the non-fixated stimulus sequence (0.661±0.019 vs. 0.528±0.016, respectively). There was also a main effect of Shift Set, *F*(2,38) = 3.64, *p* = .036, reflecting slightly decreased phase locking after the first shift set, especially in the second (0.606±0.015, 0.583±0.016, and 0.594±0.017 in shift sets 1, 2, and 3, respectively).

However, the average difference between SSVEP phase locking to the fixated and non-fixated stimuli differed across shift sets, as indicated by a Stimulus×Shift Set interaction, *F*(2,38) = 7.93, *p* = .004. The average phase locking to the fixated stimuli decreased after the first shift set (0.693±0.019, 0.642±0.021, and 0.648±0.020 in shift sets 1, 2, and 3, respectively), while the average phase locking to the non-fixated stimuli increased slightly (0.519±0.018, 0.524±0.016, and 0.540±0.019 in shift sets 1, 2, and 3, respectively). Furthermore, the difference between phase locking to the fixated and non-fixated stimuli also depended on the time until the next shift, as indicated by a Stimulus×Time interaction, *F*(120,2280) = 1.50, *p* = .036. See Figure 5. Finally, the time course of average phase locking depended marginally on Lead, F(120,2280) = 1.47, p = .049. There were no other main or interaction effects for SSVEP amplitude, all *p*>.05.

### Recognition Test Performance

To test whether elevated SSVEP amplitude predicted subsequent recognition of fixated stimuli, local amplitude maxima were identified in intervals during which amplitude exceeded 1 SD above the mean for more than two consecutive moving windows. A total of 10 such local maxima were identified for the SSVEP driven by the fixated stimulus sequence, and 12 for the SSVEP driven by the non-fixated sequence. See Figure 3. Correlations between SSVEP amplitude at the local maxima and subsequent recognition of fixated stimuli were tested using critical *p*-values of .05/10 for the SSVEP driven by the fixated sequence and .05/12 for the SSVEP driven by the non-fixated sequence. The amplitude of the SSVEP driven by the fixated stimuli predicted subsequent recognition of previously fixated stimuli, as measured by *d′* (1.38±0.23), at two points during the third shift set, approximately 2400 ms (*p* = .0018) and 400 ms (*p* = .0046) before gaze shifted (Figure 3). Recognition was not predicted by the amplitude of the SSVEP driven by the non-fixated stimuli, nor by the duration of looks.

Similar analyses were conducted for SSVEP phase delay and phase locking. For phase delay, local minima were identified in intervals during which phase delay was more than 1 SD below the mean for more than two consecutive moving windows. A total of 5 such local minima were identified for the SSVEP driven by the fixated stimulus sequence, and 4 for the SSVEP driven by the non-fixated sequence. None of the correlations between phase delay at the local minima and subsequent recognition of fixated stimuli were significant using critical *p*-values of .05/5 and .05/4, respectively. See Figure 4.

For phase locking, local maxima were identified in intervals during which phase locking exceeded 1 SD above the mean for more than two consecutive moving windows. A total of 7 such local maxima were identified for the SSVEP driven by the fixated stimulus sequence, and 6 for the SSVEP driven by the non-fixated sequence. None of the correlations between phase locking at the local maxima and subsequent recognition of fixated stimuli were significant using critical *p*-values of .05/7 and .05/6, respectively. See Figure 5.

## Discussion

Changes in attention to fixated and non-fixated stimuli in the seconds before voluntary (un-cued) shifts of gaze were revealed by steady state visual evoked potentials (SSVEPs) recorded over temporal-occipital cortex. As expected, SSVEPs driven by fixated stimuli were larger than SSVEPs driven by non-fixated stimuli. Importantly, however, the difference decreased across shift sets within blocks; the decrease was due more to increased attention to the non-fixated stimuli than to decreased attention to the fixated stimuli. In addition, there were robust fluctuations in both which were time-locked to the impending spontaneous shift of gaze and dependent on the number of shifts that had already been executed. The increase in SSVEP amplitude to the non-fixated stimuli across shift sets, and the undiminished variance of the amplitude of the SSVEPs driven by both stimulus sequences, make it unlikely that the pattern of amplitude results were due to sensory adaptation.

While phase *delay* showed no informative patterns (other than a slight decrease across shift sets), phase *locking* between the SSVEPs and the stimulus sequences that drove them exhibited a pattern of differences that was similar to the pattern in SSVEP amplitude. That is, as expected, phase locking to the fixated stimulus sequence was greater than to the non-fixated sequence, but the difference diminished across shift sets and was due to decreases in phase locking to the fixated sequence and increases in phase locking to the non-fixated sequence. Furthermore, there were systematic fluctuations in the difference that depended on the time until the next spontaneous gaze shift. The phase locking results are consistent with recent evidence that one mechanism by which attention increases SSVEP amplitude is through enhanced synchronization of the responses of visual neurons with the temporal sequence of stimuli [21].

The fact that the fluctuations in SSVEP amplitude and phase locking depended on the time until the impending spontaneous shift of gaze raises the interesting possibility that specific changes in SSVEP parameters might accurately signal gaze shifts within individuals in real time. However, substantially more data from each subject would be needed in order to address this question directly. Such data would be relevant to the use of SSVEP methods in the basic study of cognitive dynamics, especially in free looking conditions, as well the development of more powerful brain-computer interfaces.

Subsequent recognition of individual stimuli seen for 62.5 ms 1–2 times each second before participants shifted their gaze was predicted by the amplitude of the SSVEP driven by the fixated stimuli in the final shift set before recognition was tested. It is unclear whether transiently greater attention to the fixated stimuli in the final shift set directly facilitated encoding of the stimuli, or simply marked participants who remained more engaged in the task throughout the block. In any case, the timing of the intervals in the final shift set during which SSVEP amplitude predicted recognition performance (the third and last second before participants shifted gaze) may reflect the time scale of basic cognitive processes engaged by the task [38].

This study and its methods have some possible limitations. First, only two signal leads were used to detect brain electrical potentials. However, the scalp regions over which attention effects on SSVEP amplitude can be detected are broad and well-documented [14], [18], which reduces the need for multiple recording sites. Furthermore, the use of only two signal leads allows rapid preparation and very low levels of distraction for participants [39]. One of our primary goals was to demonstrate the possibility of reliable recording using minimal preparation procedures. Nevertheless, recording from multiple sites is a common procedure that permits the selection of the lead or leads with optimal signal and noise characteristics in each participant [18], [40]–[42]. Second, relatively few trials were used. Again, our interest was in studying the dynamics of attention during free looking in relatively unconstrained conditions with minimal fatigue and boredom for the participants, and the SSVEPs in the present study proved to be robust. Third, the use of SSVEPs requires the use of flickering stimuli, which certainly limits the study design choices that are available. Despite this constraint, as many as 13 commands utilizing different flicker frequencies have been reliably distinguished for BCI applications presenting an exciting practical application of this technique [43]. Finally, the recognition test used during the experiment did not include pictures from the non-fixated sequence, so no behavioral analysis of covert attention to those stimuli was possible.

The present study illustrates that rich information about the temporal course of spatial attention during free looking is available with simple methods that monitor its effects on early sensory processing on a second-by-second basis over extended intervals. This information can illuminate the dynamics of cognition in relatively natural and unconstrained conditions with minimal procedural demands. In addition, it can be used to enable communication and the control of action in situations where body movements are not possible.
